# Hypertriglyceridemic waist phenotype and risk of chronic kidney disease in community-dwelling adults aged 60 years and older in Tianjin, China: a 7-year cohort study

**DOI:** 10.1186/s12882-021-02339-5

**Published:** 2021-05-19

**Authors:** Ruping Chen, Guangshan Sun, Rui Liu, Ao Sun, Yujie Cao, Xiaojie Zhou, Sha Zhang

**Affiliations:** 1 Graduate School of Tianjin Medical University, Tianjin, 300070 China; 2 Department of Clinical Laboratory, Tianjin Union Medical Centre, Tianjin, 300121 China; 3 Tianjin Junliangcheng Hospital, Tianjin, 300301 China

**Keywords:** Hypertriglyceridemic waist phenotype, Chronic kidney disease, Chinese older adults, Community, Cohort study

## Abstract

**Background:**

The hypertriglyceridemic waist (HTGW) phenotype has been proposed to be related to the occurrence and progression of chronic kidney disease (CKD). The ageing trend of the Chinese population continues to intensify, and elderly individuals are at high risk of CKD. The purpose of this study was to investigate the cross-sectional and longitudinal associations between the HTGW phenotype and the risk of CKD by following community-dwelling adults aged 60 years and older in Tianjin, China, for 7 years.

**Methods:**

This study was an observational cohort study conducted between 2013 and 2019. Of 2050 participants aged 60 years and older who underwent an annual health examination in 2013, 1605 individuals with complete data were enrolled in the cross-sectional analysis. Among them, 1271 individuals were observed until 2019. Detailed follow-up records were available for 816 participants, of whom 600 participants without CKD at baseline were eligible for inclusion in the retrospective analysis. The HTGW phenotype was defined as a waist circumference of 90 cm or more and triglyceride concentrations of 2.0 mmol/L or more in males or a waist circumference of 85 cm or more and triglyceride concentrations of 1.5 mmol/L or more in females. CKD was defined as an estimated glomerular filtration rate (eGFR) < 60 mL/min/1.73m^2^ and/or proteinuria (urinary albumin-to-creatinine ratio (ACR) ≥ 30 mg/g). Multivariable logistic regression analyses were performed to evaluate the relationship between the HTGW phenotype and CKD.

**Results:**

In 2013, the prevalence of CKD among older adults was 31.03%, and the prevalence of CKD in the HTGW phenotype group was 37.81%. Over a 7-year observation period, 195 individuals developed CKD, with an incidence rate of 32.50%. Statistically significant associations were observed between the HTGW phenotype and CKD in older adults in both cross-sectional surveys and retrospective analyses, with odds ratios and 95% confidence intervals of 1.38 (95% CI: 1.03–1.86, *P* = 0.033) and 2.27 (95% CI: 1.30–3.97, *P* = 0.004), respectively, after adjustment for confounders.

**Conclusions:**

In this community-based cohort study, the HTGW phenotype was confirmed to be independently associated with an increased risk of prevalent and incident CKD in older adults aged 60 years and above in Tianjin, China.

**Supplementary Information:**

The online version contains supplementary material available at 10.1186/s12882-021-02339-5.

## Background

Chronic kidney disease (CKD) has become a serious public health problem with an increasing prevalence over the last decade in societies worldwide [1]. It is not only a major cause of end-stage renal disease (ESRD) but also an independent risk factor for cardiovascular disease (CVD) [2]. Zhang et al. [3] reported that the overall prevalence of CKD in Chinese adults was 10.8% in 2012, and the prevalence of CKD increased from 7.4% in women aged 18–39 years to 18.0 and 24.2% in women aged 60–69 and over 70, respectively. The progression of the decline in renal function increases with ageing [4]. Recently, a meta-analysis showed that the mean global prevalence of CKD was 13.4% [5]. The prevalence of CKD increased with age, reaching higher values among older adults. It has been reported that the prevalence of CKD in the elderly in a region of Brazil was 21.4%, and the prevalence in those aged 60–69 years, 70–79 years and ≥ 80 years was 15.9, 23.7 and 37.8%, respectively [6]. With the ageing of the population and changes in lifestyle, the risk of developing CKD among the elderly is much higher than previously reported [7]. Therefore, the identification of individuals at high risk for CKD is essential for prevention and management in older adults.

Since 2012, Kidney Disease: Improving Global Outcomes (KDIGO) has pointed out that an estimated glomerular filtration rate (eGFR) < 60 mL/min/1.73m^2^ or renal injury markers such as proteinuria (urinary albumin-to-creatinine ratio (ACR) ≥ 30 mg/g) for at least 3 months can be used as diagnostic criteria for CKD [8]. Albumin appears in the urine when the glomeruli are slightly damaged. Albuminuria is a sensitive indicator for early screening for renal injury. Elevated levels of urinary albumin are associated with an increased risk of progressive renal function loss over time. Since 2006, the establishment of health examination files for older adults aged 60 years and older has been popularized and promoted in Tianjin, China. However, in most parts of China, when a free annual health check-up for the elderly is conducted, the indicators used to detect renal injury are generally serum creatinine and urea nitrogen, and urinary albumin is excluded, which may reduce the sensitivity of CKD screening.

Currently, the hypertriglyceridemic waist (HTGW) phenotype has gained increasing attention. The concept was introduced by Lemieux et al. in 2000 as a valuable and simple tool for clinicians that required the measurement of only two components [9]. The HTGW phenotype was defined as waist circumference (WC) ≥ 90 cm and plasma triglyceride (TG) concentrations ≥ 2.0 mmol/L in males, while for females, the cut-off values were 85 cm for WC and 1.5 mmol/L for TG concentrations [9, 10]. Elevated fasting triglyceride combined with increased waist circumference is a precursor of metabolic abnormalities. In previous studies, the presence of the HTGW phenotype has been used to evaluate the risk of CVD and diabetes [11, 12]. In addition, several studies have shown the relationship between the HTGW phenotype and CKD. Previous cross-sectional research has reported that the HTGW phenotype may be a useful clinical indicator for identifying adults at high risk of CKD [13–15]. Another study pointed out that the HTGW phenotype is positively correlated with CKD among the elderly Chinese population [16]. Ramezankhani A et al. [17] found that the HTGW phenotype was associated with prevalent CKD in a cross-sectional setting, while it had no significant effect on the prediction of new-onset CKD in a prospective analysis.

To the best of our knowledge, the relationship between the HTGW phenotype and high-risk status for future CKD has not been previously assessed in adults aged 60 years and older in a developing country such as China. CKD prevention awareness is insufficient among elderly adults in developing countries. The early onset of CKD is hidden and difficult to detect. When renal function changes significantly and symptoms appear, the patient is often in the middle and late stages of renal disease, and the best opportunity for treatment has been missed. In addition, the progression of CKD leads to many complications, which will place a heavy mental and economic burden on patients. The ageing trend of the Chinese population is increasingly intensifying, and elderly individuals are at high risk of CKD. Therefore, early detection of and intervention for CKD in community-dwelling older adults and control and reduction of risk factors are conducive to delaying the progression of CKD and preventing the occurrence of ESRD. Paying close attention to waist circumference and triglyceride levels and providing timely control are effective measures for reducing the risk of CKD development. Here, we tested the hypothesis that the HTGW phenotype is related to an increased risk of CKD in community-dwelling adults aged 60 years and older in Tianjin, China. Regular monitoring of the HTGW phenotype is of great significance for the prevention of and early intervention for CKD in older adults.

## Methods

### Study design and study population

**Fig. 1:**
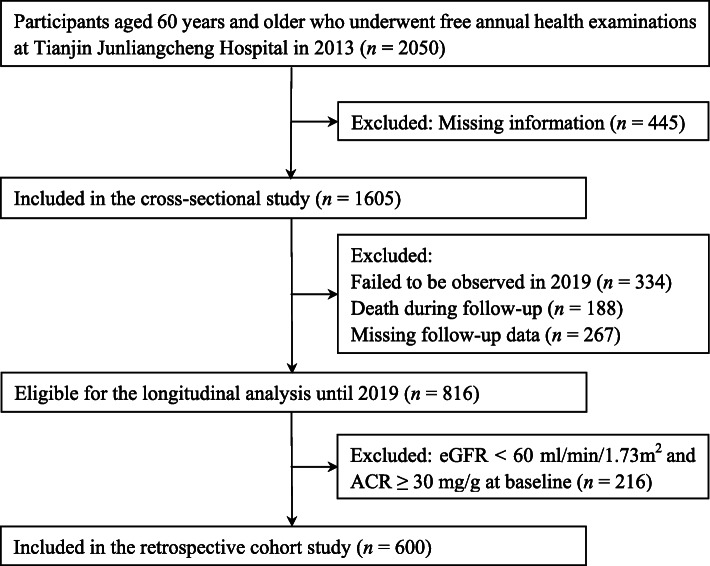
Flow chart of study enrolment

This was a community-based, cross-sectional investigation and longitudinal retrospective cohort study conducted in Tianjin, China, between April 2013 and July 2019. A natural community was under the jurisdiction of Junliangcheng Hospital of Dongli District in Tianjin. Four out of twelve villages in this community were selected by random cluster sampling. First morning urine samples were collected from 2050 participants aged ≥ 60 years who underwent free annual health examinations in 2013. Urinary albumin and urine creatinine detection was performed. Routine physical examination data were collected using electronic medical records. After those with missing information were excluded, 1605 subjects were enrolled in a cross-sectional study. Among them, 1271 subjects participated in health examinations in both 2013 and 2019, while another 334 were not examined in 2019. For the longitudinal analysis, 188 subjects were excluded due to death, and 267 subjects were excluded due to missing data. To clarify whether the HTGW phenotype was a risk factor for the future development of CKD, 216 subjects with a diagnosis of CKD at baseline were excluded. Ultimately, a sample consisting of 600 respondents with complete follow-up information over a 7-year period was eligible for a retrospective cohort study (Fig. 1). The excluded individuals had no effect on the results, indicating that there was no selection bias (Supplementary Table 1 & Supplementary Table 2). All subjects gave their written informed consent for inclusion before they participated in the study. The study was performed in accordance with the Declaration of Helsinki, and the protocol was approved by the ethics committee of Tianjin Union Medical Centre (2020-B08).

### Questionnaire survey

Standardized interviews were administered by well-trained nurses. Data on age, sex, personal history (hypertension and diabetes) and lifestyles were obtained. Current smoking was defined as one or more cigarettes per day for at least 1 year. Current drinking was defined as 50 mL or more of ethanol per day for at least 1 year. A high-salt diet was described as the self-reported consumption of too much salt (more than 6 g of salt per person per day). Regular exercise was described as performing any kind of physical activity regularly at least once a week. Hypertension was diagnosed as systolic blood pressure (SBP) ≥ 140 mmHg and/or diastolic blood pressure (DBP) ≥ 90 mmHg, a self-reported history of hypertension, or the self-reported use of antihypertensive medications [18]. Diabetes was diagnosed as fasting plasma glucose (FPG) ≥ 7.0 mmol/L, a self-reported history of diabetes, or the self-reported use of antidiabetic medications [19]. Antihypertensive treatment included oral antihypertensive drugs, such as nifedipine. Antidiabetic therapy included oral hypoglycaemic agents or subcutaneous insulin injections.

### Physical measurements

Body weight, height, waist circumference and blood pressure were obtained between 7:00 am and 9:00 am. Body weight and height were measured using a calibrated balance-beam scale and wall-mounted ruler. Body mass index (BMI) was calculated as weight in kilograms divided by the square of height in metres (kg/m^2^). Waist circumference (WC) was measured twice, and the mean value was recorded. Systolic blood pressure (SBP) and diastolic blood pressure (DBP) were read three times on the right arm with a standardized mercury sphygmomanometer with the subject in a sitting position after resting quietly for at least 5 min, and the mean value was calculated.

### Laboratory assays

Fasting (at least 10 h overnight) venous blood samples and first morning urine specimens were collected from all participants. Plasma total cholesterol (TC), triglycerides (TG), fasting plasma glucose (FPG), blood urea nitrogen (BUN), and serum creatinine (Scr) were measured on a Mindray BS-800 automatic analyser (Mindray, Shenzhen, China) with standardized reagents provided by the manufacturer. In addition, urinary albumin and urine creatinine were measured as part of the free annual health examination for older adults of all participants except those who had symptoms of urinary tract infection. The urinary albumin-to-creatinine ratio (ACR; mg/g creatinine) was then calculated. All samples were tested simultaneously under identical environmental conditions at the same clinical laboratory where the equipment was calibrated.

### Definition of CKD

According to the 2012 Kidney Disease: Improving Global Outcomes (KDIGO) Guidelines, CKD was defined as an estimated glomerular filtration rate (eGFR) < 60 mL/min/1.73m^2^ or albuminuria (urinary albumin-to-creatinine ratio (ACR) ≥ 30 mg/g) or both for at least 3 months [8]. The eGFR value was calculated using an equation from the Chinese Modification of Diet in Renal Disease (C-MDRD) study [20]: eGFR (mL/min/1.73m^2^) = 175 × (Scr)^-1.234^ × (Age)^-0.179^ × (if female, × 0.79). CKD was screened using a single measurement and medical history.

### Definition of the HTGW phenotype

The HTGW phenotype was defined as an elevated waist circumference (≥ 90 cm in males and ≥ 85 cm in females), along with elevated plasma triglyceride concentrations (≥ 2.0 mmol/L in males and ≥ 1.5 mmol/L in females) [9, 10]. The subjects were divided into four groups, classified as NWNT (normal waist-normal triglycerides), EWNT (elevated waist-normal triglycerides), NWET (normal waist-elevated triglycerides), and HTGW.

### Statistical analysis

Statistical analyses were conducted using SPSS version 21.0 software (SPSS, Inc., Chicago, IL, USA). The baseline characteristics of the four phenotype groups were compared. For continuous variables, we used means with standard deviations (SD) for normally distributed data and medians with interquartile ranges (IQR) for nonnormally distributed data, and the significance of the differences was examined by one-way analysis of variance or nonparametric test according to the data distribution. Categorical variables are described as frequencies and percentages and were compared with the chi-square test.

Multivariable logistic regression analyses were performed to identify whether the HTGW phenotype was an independent risk factor for CKD before and after adjustment for confounders. The confounders were statistically significant risk factors for CKD identified in the univariate analyses. Odds ratios (ORs) and 95% confidence intervals (CIs) were calculated. The NWNT group was considered the reference group. Model 1 was adjusted for age and sex. Model 2 was adjusted for age, sex, smoking status, alcohol intake, high-salt diet and physical activity. Model 3 was further adjusted for hypertension and diabetes. In all analyses, *P* < 0.05 indicated statistical significance.

## Results

### Characteristics of the participants in the cross-sectional study

A total of 1605 participants were enrolled in the cross-sectional study. Their ages ranged from 60 to 89 years, with an average age of 67.26 ± 5.94 years. The proportions of participants aged 60–69 years, 70–79 years and ≥ 80 years were 69.84, 26.42 and 3.74%, respectively. The ratio of males to females was 1:1.27. According to their WC and TG levels, 552 subjects were assigned to the NWNT group, 423 subjects were assigned to the EWNT group, 191 subjects were assigned to the NWET group, and 439 subjects belonged to the HTGW phenotype.

The characteristics of the participants in different groups are presented in Table 1. The NWNT group had the highest mean age and the highest percentage of males (*P* < 0.001). The HTGW phenotype group had significantly higher BMI, FPG, TG and ACR (*P* < 0.001), while the EWNT phenotype group had significantly higher WC, SBP, DBP and Scr (*P* < 0.05). The NWET phenotype group had the highest level of TC but the lowest eGFR value (*P* < 0.001). Current smoking and current drinking were most prevalent in subjects with the NWNT and EWNT phenotypes, respectively (*P* < 0.001). Subjects with the HTGW phenotype were more likely to consume a high-salt diet and have a lower frequency of regular exercise, but no significant differences were observed. Moreover, a higher prevalence of hypertension and diabetes was observed in the HTGW phenotype group (*P* < 0.001). The proportion of antihypertensive treatment and antidiabetic therapy use was also absolutely higher in the HTGW phenotype group (*P* < 0.001).

**Table 1:** Characteristics of the participants in the cross-sectional study by phenotype

Variables	Overall	NWNT	EWNT	NWET	HTGW	*P* value
	(*n* = 1605)	(*n* = 552)	(*n* = 423)	(*n* = 191)	(*n* = 439)	
Age (years)	67.26 ± 5.94	67.85 ± 6.13	67.74 ± 6.23	66.45 ± 5.60 ^*^	66.39 ± 5.38 ^*#^	< 0.001
Male (%)	708 (44.11%)	323 (58.51%)	238 (56.26%)	40 (20.94%) ^*#^	107 (24.37%) ^*#^	< 0.001
BMI (kg/m^2^)	25.03 ± 3.51	22.31 ± 2.55	27.00 ± 2.92 ^*^	23.44 ± 2.16 ^*#^	27.24 ± 2.74 ^*§^	< 0.001
WC (cm)	87.05 ± 8.94	79.46 ± 5.97	93.75 ± 5.74 ^*^	80.35 ± 4.70 ^#^	93.05 ± 5.92 ^*§^	< 0.001
SBP (mmHg)	138.54 ± 19.08	136.44 ± 18.70	141.11 ± 17.82 ^*^	136.79 ± 21.98 ^#^	139.45 ± 19.07 ^*^	0.001
DBP (mmHg)	82.35 ± 9.82	81.49 ± 9.95	84.10 ± 9.84 ^*^	81.55 ± 9.78 ^#^	82.09 ± 9.44 ^#^	< 0.001
FPG (mmol/L)	5.26 ± 1.36	5.00 ± 1.11	5.27 ± 1.18 ^*^	5.22 ± 1.36	5.60 ± 1.69 ^*#§^	< 0.001
TC (mmol/L)	5.21 ± 0.97	5.00 ± 0.93	5.04 ± 0.92	5.55 ± 0.87 ^*#^	5.50 ± 0.99 ^*#^	< 0.001
TG (mmol/L)	1.72 ± 1.09	1.08 ± 0.35	1.22 ± 0.34 ^*^	2.37 ± 0.99 ^*#^	2.72 ± 1.30 ^*#^	< 0.001
BUN (mmol/L)	6.08 ± 2.64	6.14 ± 2.22	6.16 ± 2.88	5.67 ± 1.59 ^*#^	6.11 ± 3.19	0.151
Scr (mmol/L)	78.73 ± 12.79	79.21 ± 11.81	79.76 ± 13.16	76.97 ± 11.70 ^*#^	77.91 ± 13.95 ^#^	0.030
eGFR (ml/min/1.73m^2^)	85.82 ± 14.29	87.65 ± 13.36	86.86 ± 14.85	83.40 ± 12.51 ^*#^	83.58 ± 15.17 ^*#^	< 0.001
ACR (mg/g)	15.33 (8.31 ~ 36.32)	13.23 (7.54 ~ 29.89)	14.16 (7.95 ~ 32.65)	18.38 (9.79 ~ 43.38) ^*^	19.56 (9.87 ~ 49.62) ^*#^	< 0.001
Current smoking (%)	613 (38.19%)	247 (44.75%)	137 (32.39%) ^*^	74 (38.74%)	155 (35.31%) ^*^	< 0.001
Current drinking (%)	381 (23.74%)	167 (30.25%)	142 (33.57%)	17 (8.90%) ^*#^	55 (12.53%) ^*#^	< 0.001
High-salt diet (%)	564 (35.14%)	198 (35.87%)	143 (33.81%)	64 (33.51%)	159 (36.22%)	0.824
Regular exercise (%)	957 (59.63%)	339 (61.41%)	253 (59.81%)	111 (58.12%)	254 (57.86%)	0.685
Hypertension (%)	912 (56.82%)	251 (45.47%)	263 (62.17%) ^*^	112 (58.64%) ^*^	286 (65.15%) ^*^	< 0.001
Antihypertensive treatment (%)	891 (55.51%)	241 (43.66%)	257 (60.76%) ^*^	110 (57.59%) ^*^	283 (64.46%) ^*^	< 0.001
Diabetes (%)	211 (13.15%)	46 (8.33%)	56 (13.24%)	25 (13.09%)	84 (19.13%) ^*^	< 0.001
Antidiabetic therapy (%)	194 (12.09%)	44 (7.97%)	49 (11.58%)	22 (11.52%)	79 (18.00%) ^*#^	< 0.001

Abbreviations: *NWNT* Normal waist-normal triglycerides; *EWNT* Elevated waist-normal triglycerides; *NWET* Normal waist-elevated triglycerides; *HTGW* Hypertriglyceridemic waist; *BMI* Body mass index; *WC* Waist circumference; *SBP* Systolic blood pressure; *DBP* Diastolic blood pressure; *FPG* Fasting plasma glucose; *TC* Total cholesterol; *TG* Triglycerides; *BUN* Blood urea nitrogen; *Scr* Serum creatinine; *eGFR* Estimated glomerular filtration rate; *ACR* Albumin-to-creatinine ratio

Data are expressed as the mean ± standard deviation, median (interquartile range), or frequency (percentage).

*P* values were calculated using one-way analysis of variance or non-parametric tests for continuous variables and the chi-square test for categorical variables.

^*^ Compared with the NWNT group, *P <* 0.05

^#^ Compared with the EWNT group, *P <* 0.05

^§^ Compared with the NWET group, *P <* 0.05

### Prevalence of CKD in different phenotype groups

**Fig. 2:**
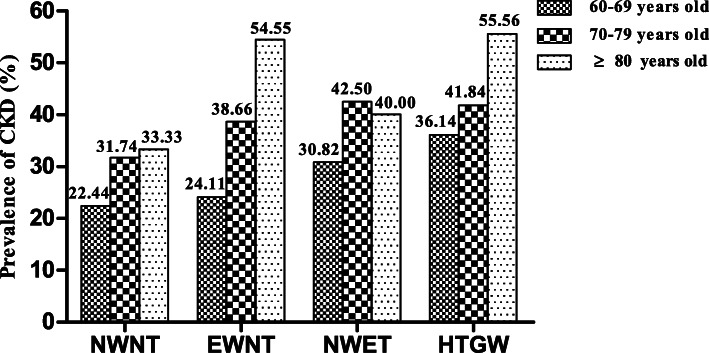
Prevalence of CKD in 1605 subjects in 2013 based on HTGW phenotype. Abbreviations: NWNT, normal waist-normal triglycerides; EWNT, elevated waist-normal triglycerides; NWET, normal waist-elevated triglycerides; HTGW, hypertriglyceridemic waist

The prevalence of CKD was 31.03% among community-dwelling adults aged 60 years and older in 2013. Subjects in the HTGW phenotype group had a significantly higher prevalence of CKD than those in the NWNT, EWNT, and NWET groups; the prevalences were 37.81, 25.72, 29.79 and 33.51%, respectively (*P* < 0.001). We divided the subjects by age group. The prevalence of CKD for each phenotype according to age group is shown in Fig. 2. The prevalence of CKD shows an increasing trend with age in each phenotype group, which supports the importance of investigating the relationship between the HTGW phenotype and the risk of CKD in older adults.

### Association of the HTGW phenotype with prevalent CKD

The logistic regression models displayed the relationships between the four phenotypes and prevalent CKD in the cross-sectional analysis (Table 2). The HTGW phenotype was significantly associated with prevalent CKD, with the NWNT phenotype serving as a reference in the unadjusted model (OR = 1.76, 95% CI: 1.34–2.30, *P* < 0.001). In addition, for the subjects with the NWET phenotype, the OR value for CKD was 1.46 (95% CI: 1.02–2.08, *P* = 0.039). After adjusting for age and sex (Model 1), the association of the HTGW phenotype with CKD was attenuated but remained significant (OR = 1.62, 95% CI: 1.22–2.15, *P* = 0.001). After adjusting for age, sex, smoking status, alcohol intake, high-salt diet, and physical activity (Model 2), there was still a significant association between the HTGW phenotype and CKD (OR = 1.62, 95% CI: 1.22–2.17, *P* = 0.001). After further adjustment for hypertension and diabetes (Model 3), subjects with the HTGW phenotype had a 1.38-fold increased risk of CKD (OR = 1.38, 95% CI: 1.03–1.86, *P* = 0.033).

**Table 2:** Multivariable logistic regression models of prevalent CKD by HTGW phenotype in the cross-sectional analysis

Phenotype	Unadjusted		Model 1		Model 2		Model 3	
	OR (95%CI)	*P* value	OR (95%CI)	*P* value	OR (95%CI)	*P* value	OR (95%CI)	*P* value
NWNT	1.00(reference)	–	1.00(reference)	–	1.00(reference)	–	1.00(reference)	–
EWNT	1.23 (0.92–1.63)	0.159	1.22 (0.92–1.63)	0.172	1.18 (0.88–1.57)	0.264	1.06 (0.79–1.43)	0.681
NWET	1.46 (1.02–2.08)	0.039	1.31 (0.91–1.90)	0.149	1.33 (0.92–1.92)	0.136	1.19 (0.82–1.74)	0.370
HTGW	1.76 (1.34–2.30)	< 0.001	1.62 (1.22–2.15)	0.001	1.62 (1.22–2.17)	0.001	1.38 (1.03–1.86)	0.033

Model 1: Adjusted for age, sex.

Model 2: Adjusted for age, sex, smoking status, alcohol intake, high-salt diet, physical activity.

Model 3: Adjusted for the above + hypertension, diabetes.

### Baseline characteristics of participants in the retrospective cohort study

Table 3 presents a summary of the characteristics of 600 respondents aged 66–91 years without CKD at baseline who completed a 7-year follow-up. A total of 212 subjects were assigned to the NWNT group, 153 subjects were assigned to the EWNT group, 74 subjects were assigned to the NWET group, and 161 subjects belonged to the HTGW phenotype group. The differences among the four groups were analysed in detail. The HTGW phenotype presented the highest BMI, WC, SBP, FPG, TG, and ACR (*P* < 0.05). In addition, subjects with the HTGW phenotype also had a significantly higher prevalence of hypertension and a higher percentage of antihypertensive treatment use (*P* < 0.001).

**Table 3:** Baseline characteristics of the 600 participants in the retrospective cohort study by phenotype

Variables	Overall	NWNT	EWNT	NWET	HTGW	*P* value
	(*n* = 600)	(*n* = 212)	(*n* = 153)	(*n* = 74)	(*n* = 161)	
Age (years)	65.82 ± 4.74	66.42 ± 5.21	65.81 ± 4.77	64.95 ± 3.99 ^*^	65.45 ± 4.31	0.078
Male (%)	296 (49.33%)	140 (66.04%)	88 (57.52%)	18 (24.32%) ^*#^	50 (31.06%) ^*#^	< 0.001
BMI (kg/m^2^)	24.95 ± 3.30	22.56 ± 2.43	26.61 ± 2.64 ^*^	23.27 ± 2.44 ^*#^	27.28 ± 2.58 ^*#§^	< 0.001
WC (cm)	87.01 ± 8.21	80.43 ± 5.64	92.78 ± 5.09 ^*^	80.68 ± 4.42 ^#^	93.08 ± 5.67 ^*§^	< 0.001
SBP (mmHg)	136.33 ± 17.73	134.30 ± 16.98	138.40 ± 15.11^*^	132.73 ± 22.45 ^#^	138.67 ± 18.14 ^*§^	0.013
DBP (mmHg)	82.24 ± 9.31	81.43 ± 9.97	83.94 ± 8.60 ^*^	80.81 ± 8.44 ^#^	82.35 ± 9.28	0.037
FPG (mmol/L)	5.13 ± 1.04	4.90 ± 0.69	5.26 ± 1.17 ^*^	5.12 ± 1.30	5.31 ± 1.11 ^*^	0.002
TC (mmol/L)	5.19 ± 0.91	5.00 ± 0.87	5.03 ± 0.85	5.49 ± 0.74 ^*#^	5.44 ± 1.00 ^*#^	< 0.001
TG (mmol/L)	1.70 ± 1.08	1.09 ± 0.37	1.21 ± 0.35	2.22 ± 0.63 ^*#^	2.76 ± 1.38 ^*#^	< 0.001
BUN (mmol/L)	5.98 ± 3.00	6.18 ± 2.99	6.23 ± 4.32	5.47 ± 1.65	5.71 ± 1.60	0.142
Scr (mmol/L)	77.17 ± 10.74	78.12 ± 9.87	77.40 ± 12.09	75.58 ± 9.92	76.44 ± 10.78	0.088
eGFR (ml/min/1.73m^2^)	88.69 ± 12.36	90.59 ± 12.87	90.37 ± 12.64	85.69 ± 10.56 ^*#^	85.97 ± 11.48 ^*#^	< 0.001
ACR (mg/g)	10.60 (6.72 ~ 16.48)	9.04 (5.95 ~ 15.22)	10.20 (6.62 ~ 15.22)	11.90 (7.23 ~ 18.59)	12.66 (7.83 ~ 19.57) ^*#^	0.001
Current smoking (%)	231 (38.50%)	96 (45.28%)	49 (32.03%)	30 (40.54%)	56 (34.78%)	0.049
Current drinking (%)	157 (26.17%)	74 (34.91%)	43 (28.10%)	9 (12.16%) ^*#^	31 (19.25%) ^*^	< 0.001
High-salt diet (%)	230 (38.33%)	76 (35.85%)	61 (39.87%)	31 (41.89%)	62 (38.51%)	0.771
Regular exercise (%)	351 (58.50%)	129 (60.85%)	83 (54.25%)	40 (54.05%)	99 (61.49%)	0.421
Hypertension (%)	317 (52.83%)	89 (41.98%)	84 (54.90%)	40 (54.05%)	104 (64.60%) ^*^	< 0.001
Antihypertensive treatment (%)	311 (51.83%)	87 (41.04%)	82 (53.59%)	39 (52.70%)	103 (63.98%) ^*^	< 0.001
Diabetes (%)	61 (10.17%)	13 (6.13%)	17 (11.11%)	8 (10.81%)	23 (14.29%)	0.062
Antidiabetic therapy (%)	55 (9.17%)	12 (5.66%)	13 (8.50%)	8 (10.81%)	22 (13.66%)	0.058

Abbreviations: *NWNT* Normal waist-normal triglycerides; *EWNT* Elevated waist-normal triglycerides; *NWET* Normal waist-elevated triglycerides; *HTGW* Hypertriglyceridemic waist; *BMI* Body mass index; *WC* Waist circumference; *SBP* Systolic blood pressure; *DBP* Diastolic blood pressure; *FPG* Fasting plasma glucose; *TC* Total cholesterol; *TG* Triglycerides; *BUN* Blood urea nitrogen; *Scr* Serum creatinine; *eGFR* Estimated glomerular filtration rate; *ACR* Albumin-to-creatinine ratio

Data are expressed as the mean ± standard deviation, median (interquartile range), or frequency (percentage).

*P* values were calculated using one-way analysis of variance or non-parametric tests for continuous variables and the chi-square test for categorical variables.

^*^ Compared with the NWNT group, *P <* 0.05

^#^ Compared with the EWNT group, *P <* 0.05

^§^ Compared with the NWET group, *P <* 0.05

### Association between the HTGW phenotype and incident CKD

Of the 600 respondents without CKD at baseline, 195 incident cases of CKD were observed in 2019. The cumulative incidence of CKD was 32.50% from 2013 to 2019. Cases of new-onset CKD in subjects with the NWNT, EWNT, NWET, and HTGW phenotypes numbered 62 (29.25%), 43 (28.10%), 42 (56.76%), and 48 (29.81%), respectively. As shown in Table 4, the HTGW phenotype was significantly associated with incident CKD when compared with the NWNT group in the unadjusted model (OR = 1.94, 95% CI: 1.13–3.32, *P* = 0.016). The OR value for developing CKD was 1.97 (95% CI: 1.15–3.40, *P* = 0.014) after adjusting for age and sex (Model 1), and it increased to 2.11 (95% CI: 1.22–3.65, *P* = 0.008) after additional adjustment for smoking status, alcohol intake, high-salt diet, and physical activity (Model 2). The association between the HTGW phenotype and risk of incident CKD remained significant (OR = 2.27, 95% CI: 1.30–3.97, *P* = 0.004) after further adjustment for potential factors, particularly hypertension and diabetes (Model 3).

**Table 4:** The retrospective associations between HTGW phenotype and incident CKD during the follow-up period

Phenotype	Unadjusted		Model 1		Model 2		Model 3	
	OR (95%CI)	*P* value	OR (95%CI)	*P* value	OR (95%CI)	*P* value	OR (95%CI)	*P* value
NWNT	1.00(reference)	–	1.00(reference)	–	1.00(reference)	–	1.00(reference)	–
EWNT	0.91 (0.58–1.44)	0.697	1.06 (0.66–1.70)	0.815	1.10 (0.68–1.77)	0.702	1.20 (0.73–1.95)	0.473
NWET	0.87 (0.53–1.42)	0.581	1.02 (0.61–1.70)	0.941	1.01 (0.60–1.69)	0.976	1.07 (0.63–1.80)	0.811
HTGW	1.94 (1.13–3.32)	0.016	1.97 (1.15–3.40)	0.014	2.11 (1.22–3.65)	0.008	2.27 (1.30–3.97)	0.004

Model 1: Adjusted for age, sex.

Model 2: Adjusted for age, sex, smoking status, alcohol intake, high-salt diet, physical activity.

Model 3: Adjusted for the above + hypertension, diabetes.

## Discussion

A cross-sectional survey and 7-year longitudinal retrospective analysis were conducted to investigate the effect of the HTGW phenotype on CKD in older adults. This 7-year cohort study revealed that the HTGW phenotype was significantly associated with an increased risk of both prevalent and incident CKD among community-dwelling adults aged 60 years and older in Tianjin, China. Subjects with the HTGW phenotype were 1.38-fold and 2.27-fold more likely to have a higher prevalence and incidence of CKD, respectively, than those with the normal phenotype. Meanwhile, the associations persisted after adjustment for possible confounders such as age, sex, smoking status, alcohol intake, high-salt diet, physical activity, hypertension, and diabetes. These results suggest that the HTGW phenotype is an independent risk factor for CKD in elderly individuals.

With the ageing of the population and changes in lifestyle, the spectrum of human diseases has changed dramatically. The prevalence of obesity is growing at an alarming rate, rapidly becoming a serious global threat. Obesity is related to increased health risks for many chronic diseases, such as cardiovascular events, diabetes, and CKD [21–23]. Clinically, there are many effective indicators that can be reflect obesity. BMI is a widely accepted indicator for the standardized definition of overweight and obesity. In addition, waist circumference, waist-to-hip ratio, waist-to-height ratio, and waist-by-height ^0.5^ ratio are relevant to abdominal adiposity [24]. A few studies have proven that visceral obesity strongly correlates with metabolic abnormalities [25, 26]. Published data have signified that the HTGW phenotype can be used in clinical practice to investigate CVD risk and visceral adipose tissue in individuals [11, 27]. The HTGW phenotype has been corroborated to be a surrogate marker for predicting visceral obesity, which is associated with metabolic alterations [28]. Moreover, it has been reported that the HTGW phenotype is significantly associated with early diabetic nephropathy. Type 2 diabetic patients with the HTGW phenotype are more vulnerable to kidney injury [29].

In recent years, CKD has received increasing attention in older adults because of its high prevalence and harmfulness. Several studies have focused on the relationship between the HTGW phenotype and CKD. A cross-sectional study has shown that the prevalence of CKD in individuals with the NWNT, NWET/EWNT and HTGW phenotypes is 9.4, 16.6 and 26.6%, respectively, in the Chinese population aged 40 years and older. The HTGW phenotype is closely related to the presence of CKD. After adjustment for potential confounders, individuals with the HTGW phenotype were 2.09 times as likely to have CKD than those with the NWNT phenotype [13]. Another study investigated 2102 urban Chinese participants aged 60–95 years. The CKD prevalence in those with the NWNT, NWET, EWNT, and HTGW phenotypes was 9.0, 3.8, 12.8, and 17.0%, respectively. It has been shown that participants with the HTGW phenotype have 95% higher odds of CKD than those in the NWNT group [16].

Older adults are at high risk for CKD [4]. To further explore the association between the HTGW phenotype and CKD in Chinese elderly individuals, 1605 participants aged 60 years and older were surveyed in 2013. Our findings suggest that participants with the HTGW phenotype had a significantly larger proportion of metabolic risk factors than participants in the other groups, including a higher BMI, greater WC, higher blood pressure, and higher levels of FPG, TC, and TG. Previous studies have reported similar results [13–16]. In the cross-sectional study, the overall prevalence of CKD was 31.03% in older adults. The prevalence of CKD in the HTGW phenotype group was 37.81%. In addition, in our retrospective cohort of 600 subjects without CKD at baseline, 32.50% developed CKD over 7 years of follow-up. The incidence of CKD was also slightly higher. Moreover, multivariable logistic regression analyses demonstrated that the HTGW phenotype was significantly associated with CKD in older adults. First, it was observed that subjects with the HTGW phenotype had a 38% increased risk of prevalent CKD (OR = 1.38, 95% CI: 1.03–1.86, *P =* 0.033) compared with subjects with normal WC and TG in the cross-sectional survey. A similar trend was found in Ramezankhani A’s research [17]. In addition, the present study provided evidence that the odds ratio for the future risk of developing CKD in participants with the HTGW phenotype was 2.27 (OR = 2.27, 95% CI: 1.30–3.97, *P =* 0.004) after adjustment for confounders, including hypertension and diabetes, in retrospective cohort analysis.

Excessive waist circumference reduces the body’s mobilization and utilization of free fatty acids, leading to the accumulation of free fatty acids in the blood and an increased volume of blood lipids. Controlling waist circumference at an appropriate level helps reduce the prevalence of hypertriglyceridemia. In our previous study, elevated waist circumference and high levels of triglycerides were strongly correlated with the progression of kidney injury [30]. The following are several potential mechanisms for this relationship: It has been confirmed that oxidative stress and chronic inflammation caused by upregulation of proinflammatory adipokines and cytokines often occur in visceral adipose tissue [31]. Visceral obesity and elevated TG levels may lead to fat accumulation in ectopic tissues. If ectopic accumulation of fat occurs in the kidneys, it may cause renal compression, which exerts an adverse influence on renal haemodynamic patterns [32]. Generally, the degree of hypertriglyceridemia is directly proportional to the severity of renal insufficiency [33]. This may explain to some extent why the HTGW phenotype is an important risk factor for CKD. Given that WC measurements and fasting plasma triglyceride concentrations are readily available and relatively inexpensive to collect, we speculate that the HTGW phenotype is an advantageous indicator for CKD screening and may play a vital role in early intervention for the prevention of CKD in older adults.

The main strengths of our study include its community-based longitudinal design and the inclusion of older adults. Available data were collected in a standardized manner. Several limitations of our study should be mentioned. First, definitions of the HTGW phenotype are inconsistent, and cut-offs need to be further validated for diverse ethnic groups, for both genders and across different age groups. Second, our follow-up observation was based on single measurements of serum creatinine, urinary albumin and creatinine at annual health examinations. However, the diagnosis of CKD usually requires more than 3 months of observation, which might limit the accuracy of our results. Third, information on lifestyles was collected via questionnaires. There might be potential recall bias in these variables. Although the analysis adjusted for multiple variables, some noteworthy confounders not obtained might exist to explain the associations. In addition, nearly half of the older adults were lost during follow-up, although a sensitivity analysis showed that the subjects who were excluded due to incomplete data had minimal effect on the results (Supplementary Table 3 & Supplementary Table 4). Finally, all participants were from the same geographical area. Hence, the results should be generalized to other populations with caution. The development of CKD in older adults is truly a major public health concern. Further investigations are essential to confirm our conclusions.

## Conclusions

In summary, we confirmed that there were strong associations between the HTGW phenotype and CKD in both cross-sectional and retrospective studies of community-dwelling older adults aged 60 years and above in Tianjin, China. Participants with the HTGW phenotype were more likely to develop CKD than those with the NWNT phenotype. Consequently, the HTGW phenotype, a convenient indicator of visceral obesity, can serve as an independent predictor for elderly individuals at high risk of CKD occurrence and progression. This suggests that maintaining normal waist circumference and triglyceride levels are ways to protect renal function in older adults.

## Supplementary Information

**Additional file 1. **For the longitudinal analysis of the association between the HTGW phenotype and CKD, 816 subjects with complete follow-up data were eligible, while 789 subjects without complete follow-up data were excluded. There was no significant difference in baseline characteristics between the two groups (**Supplementary Table 1**). To clarify whether the HTGW phenotype was a risk factor for the future development of CKD, 216 participants with a diagnosis of CKD at baseline were excluded. In general, four groups of subjects were excluded: those who failed to be observed in 2019 (*n* = 334), those who died during follow-up (*n* = 188), those who were missing follow-up data (*n* = 267) and those who had CKD at baseline (*n* = 216). The chi-square test was used to compare the distribution of NWNT, EWNT, NWET and HTGW among the four excluded groups and the complete data group. As shown in **Supplementary Table 2**, there was no significant difference in the distribution of the four phenotypes among the group that failed to be observed in 2019, the group with missing follow-up data and the group with complete data. However, the proportions of NWNT and HTGW in subjects who died during follow-up were significantly different from those proportions in the complete data group. The main causes of death of 188 individuals were tumours and cardio-cerebrovascular diseases, which could explain the differences to some extent. Because nearly half of the older adults were lost to follow-up, a sensitivity analysis was necessary to determine the changes in associations using different approaches. Table 4 in the results section shows the retrospective associations between the HTGW phenotype and incident CKD during follow-up in 600 respondents without CKD at baseline. Due to the high number of lost cases, a sensitivity analysis was conducted. **Supplementary Table 3** shows the results assuming that all the lost cases developed CKD. **Supplementary Table 4** shows the results assuming that none of the lost cases developed CKD. The risk of those with the HTGW phenotype developing incident CKD was calculated by multivariable logistic regression analyses. Kendall’s W test was used to analyse the consistency of the relationship between HTGW phenotype and the risk of incident CKD in the three tables. Kendall’s W coefficient was 0.911 (*P* = 0.042). Thus, excluding subjects with incomplete data had little effect on the conclusion.

## Data Availability

The datasets used during the current study are available from the corresponding author on reasonable request.

## Abbreviations

HTGW: Hypertriglyceridemic waist
CKD: Chronic kidney disease
eGFR: Estimated glomerular filtration rate
ACR: Albumin-to-creatinine ratio
ESRD: End-stage renal disease
CVD: Cardiovascular disease
KDIGO: Kidney disease: improving global outcomes
WC: Waist circumference
TG: Triglyceride
SBP: Systolic blood pressure
DBP: Diastolic blood pressure
FPG: Fasting plasma glucose
BMI: Body mass index
TC: Total cholesterol
BUN: Blood urea nitrogen
Scr: Serum creatinine
C-MDRD: Chinese-modification of diet renal disease
NWNT: Normal waist-normal triglycerides
EWNT: Elevated waist-normal triglycerides
NWET: Normal waist-elevated triglycerides
SD: Standard deviation
IQR: Interquartile range
OR: Odds ratio
CI: Confidence interval
