# Supplementary material for: Hypertriglyceridemic waist phenotype and risk of chronic kidney disease in community-dwelling adults aged 60 years and older in Tianjin, China: a 7-year cohort study
Source: BMC Nephrol. 2021 May 19;22:182. doi: 10.1186/s12882-021-02339-5 (PMC8132422; doi:10.1186/s12882-021-02339-5)
Supplement: Supplementary file 1 — Additional file 1. For the longitudinal analysis of the association between the HTGW phenotype and CKD, 816 subjects with complete follow-up data were eligible, while 789 subjects without complete follow-up data were excluded. There was no significant difference in baseline characteristics between the two groups (Supplementary Table 1). To clarify whether the HTGW phenotype was a risk factor for the future development of CKD, 216 participants with a diagnosis of CKD at baseline were excluded. In general, four groups of subjects were excluded: those who failed to be observed in 2019 (n = 334), those who died during follow-up (n = 188), those who were missing follow-up data (n = 267) and those who had CKD at baseline (n = 216). The chi-square test was used to compare the distribution of NWNT, EWNT, NWET and HTGW among the four excluded groups and the complete data group. As shown in Supplementary Table 2, there was no significant difference in the distribution of the four phenotypes among the group that failed to be observed in 2019, the group with missing follow-up data and the group with complete data. However, the proportions of NWNT and HTGW in subjects who died during follow-up were significantly different from those proportions in the complete data group. The main causes of death of 188 individuals were tumours and cardio-cerebrovascular diseases, which could explain the differences to some extent. Because nearly half of the older adults were lost to follow-up, a sensitivity analysis was necessary to determine the changes in associations using different approaches. Table 4 in the results section shows the retrospective associations between the HTGW phenotype and incident CKD during follow-up in 600 respondents without CKD at baseline. Due to the high number of lost cases, a sensitivity analysis was conducted. Supplementary Table 3 shows the results assuming that all the lost cases developed CKD. Supplementary Table 4 shows the results assuming that none of the l [file 12882_2021_2339_MOESM1_ESM.docx]

| **Supplementary Table 1** Baseline characteristics of subjects with and without complete follow-up data | | | |
| --- | --- | --- | --- |
| Variables | Complete follow-up data | | *P* value |
|  | With (n=816) | Without (n=789) |  |
| Age (years) | 66.82±4.85 | 67.74±6.57 | 0.061 |
| Male (%) | 366(44.85%) | 342(43.35%) | 0.543 |
| BMI (kg/m^2^) | 25.12±3.32 | 24.94±3.70 | 0.288 |
| WC (cm) | 87.34±8.40 | 86.75±9.47 | 0.184 |
| SBP (mmHg) | 137.88±18.06 | 139.19±20.09 | 0.172 |
| DBP (mmHg) | 82.72±9.54 | 81.98±10.09 | 0.133 |
| FPG (mmol/L) | 5.19±1.16 | 5.30±1.53 | 0.056 |
| TC (mmol/L) | 5.20±0.93 | 5.22±1.00 | 0.679 |
| TG (mmol/L) | 1.78±1.14 | 1.65±1.03 | 0.071 |
| BUN (mmol/L) | 6.03±3.36 | 6.13±1.60 | 0.436 |
| Scr (mmol/L) | 78.71±12.27 | 78.76±13.25 | 0.133 |
| eGFR (ml/min/1.73m^2^) | 86.40±13.04 | 84.86±15.35 | 0.090 |
| ACR (mg/g) | 14.62(7.94~31.28) | 15.81(8.66~40.34) | 0.082 |
| Current smoking (%) | 301(36.89%) | 311(39.42%) | 0.297 |
| Current drinking (%) | 202(24.75%) | 179(22.69%) | 0.330 |
| High-salt diet (%) | 298(36.52%) | 266(33.71%) | 0.064 |
| Regular exercise (%) | 475(58.21%) | 482(61.09%) | 0.240 |
| Hypertension (%) | 455(55.76%) | 456(57.79%) | 0.411 |
| Antihypertensive treatment (%) | 446(54.66%) | 444(56.27%) | 0.515 |
| Diabetes (%) | 97(11.89%) | 114(14.45%) | 0.129 |
| Antidiabetic therapy (%) | 92(11.27%) | 102(12.93%) | 0.108 |
| Abbreviations: BMI, body mass index; WC, waist circumference; SBP, systolic blood pressure; DBP, diastolic blood pressure; FPG, fasting plasma glucose; TC, total cholesterol; TG, triglycerides; BUN, blood urea nitrogen; Scr, serum creatinine; eGFR, estimated glomerular filtration rate; ACR, albumin-to-creatinine ratio. | | | |
|  |  |  |  |
|  |  |  |  |
|  |  |  |  |
| Data are expressed as the mean ± standard deviation, median (interquartile range) or frequency (percentage). | | | |
| *P* values were calculated with the *t*-test or non-parametric test for continuous variables and chi-square test for categorical variables. | | | |

| **Supplementary Table 2** The distribution of four phenotypes among the excluded groups and the complete data group | | | | | |
| --- | --- | --- | --- | --- | --- |
|  | Complete data group (n=816) | Failed to be observed in 2019 (n=334) | Died during follow-up (n=188) | Missing follow-up data (n=267) | CKD at baseline (n=216) |
| NWNT | 272(33.33%) | 112(33.53%) | 87(46.28%) ^*^ | 81(30.34%) | 60(27.78%) |
| EWNT | 202(24.75%) | 89(26.65%) | 51(27.13%) | 82(30.71%) | 49(22.69%) |
| NWET | 100(12.25%) | 41(12.28%) | 18(9.57%) | 32(11.99%) | 26(12.04%) |
| HTGW | 242(29.66%) | 92(27.54%) | 32(17.02%) ^*^ | 72(26.97%) | 81(37.50%) ^*^ |
| Abbreviations: NWNT, normal waist-normal triglycerides; EWNT, increased waist-normal triglycerides; NWET, normal waist-elevated triglycerides; HTGW, hypertriglyceridemic waist. | | | | | |
| Data are expressed as frequency (percentage). | | | | | |
| Statistical analysis was performed with the chi-square test. | | | | | |
| ^*^ Compared with the complete data group, *P* < 0.001. | | | | | |

| **Supplementary Table 3** The retrospective associations between HTGW phenotype and incident CKD during follow-up | | | | | | | | |
| --- | --- | --- | --- | --- | --- | --- | --- | --- |
| Phenotype | Unadjusted | | Model 1 | | Model 2 | | Model 3 | |
|  | OR(95%CI) | *P* value | OR(95%CI) | *P* value | OR(95%CI) | *P* value | OR(95%CI) | *P* value |
| NWNT | 1.00(reference) | - | 1.00(reference) | - | 1.00(reference) | - | 1.00(reference) | - |
| EWNT | 0.91(0.59-1.41) | 0.672 | 1.14(0.71-1.81) | 0.588 | 1.18(0.74-1.89) | 0.490 | 1.28(0.79-2.07) | 0.311 |
| NWET | 0.87(0.54-1.40) | 0.563 | 1.08(0.65-1.80) | 0.754 | 1.07(0.64-1.79) | 0.786 | 1.13(0.68-1.90) | 0.638 |
| HTGW | 2.01(1.19-3.38) | <0.001 | 1.92(1.13-3.29) | 0.017 | 2.09(1.21-3.60) | 0.005 | 2.23(1.29-3.87) | 0.002 |
| Model 1: Adjusted for age, sex. | | | | | | | | |
| Model 2: Adjusted for age, sex, smoking status, alcohol intake, high-salt diet, physical activity. | | | | | | | | |
| Model 3: Adjusted for the above + hypertension, diabetes. | | | | | | | | |

| **Supplementary Table 4** The retrospective associations between HTGW phenotype and incident CKD during follow-up | | | | | | | | |
| --- | --- | --- | --- | --- | --- | --- | --- | --- |
| Phenotype | Unadjusted | | Model 1 | | Model 2 | | Model 3 | |
|  | OR(95%CI) | *P* value | OR(95%CI) | *P* value | OR(95%CI) | *P* value | OR(95%CI) | *P* value |
| NWNT | 1.00(reference) | - | 1.00(reference) | - | 1.00(reference) | - | 1.00(reference) | - |
| EWNT | 0.95(0.64-1.42) | 0.801 | 0.95(0.60-1.50) | 0.814 | 0.97(0.61-1.54) | 0.896 | 1.05(0.65-1.67) | 0.852 |
| NWET | 0.92(0.60-1.41) | 0.692 | 0.93(0.57-1.53) | 0.779 | 0.92(0.56-1.52) | 0.749 | 0.97(0.59-1.60) | 0.893 |
| HTGW | 1.58(0.96-2.61) | 0.073 | 1.96(1.15-3.36) | 0.019 | 2.05(1.19-3.52) | 0.010 | 2.18(1.26-3.77) | 0.006 |
| Model 1: Adjusted for age, sex. | | | | | | | | |
| Model 2: Adjusted for age, sex, smoking status, alcohol intake, high-salt diet, physical activity. | | | | | | | | |
| Model 3: Adjusted for the above + hypertension, diabetes. | | | | | | | | |
